# An achromatic metasurface waveguide for augmented reality displays

**DOI:** 10.1038/s41377-025-01761-w

**Published:** 2025-02-25

**Authors:** Zhongtao Tian, Xiuling Zhu, Philip A. Surman, Zhidong Chen, Xiao Wei Sun

**Affiliations:** 1 Institute of Nanoscience and Applications, and Department of Electrical and Electronic Engineering, Southern University of Science and Technology, Shenzhen, 518055 China; 2 PengCheng Laboratory, Shenzhen, 518055 China

**Keywords:** Displays, Photonic devices

## Abstract

Augmented reality (AR) displays are emerging as the next generation of interactive platform, providing deeper human-digital interactions and immersive experiences beyond traditional flat-panel displays. Diffractive waveguide is a promising optical combiner technology for AR owing to its potential for the slimmest geometry and lightest weight. However, severe chromatic aberration of diffractive coupler has constrained widespread adoption of diffractive waveguide. Wavelength-dependent light deflection, caused by dispersion in both in-coupling and out-coupling processes, results in limited full-color field of view (FOV) and nonuniform optical responses in color and angular domains. Here we introduce an innovative full-color AR system that overcomes this long-standing challenge of chromatic aberration using a combination of inverse-designed metasurface couplers and a high refractive index waveguide. The optimized metasurface couplers demonstrate true achromatic behavior across the maximum FOV supported by the waveguide (exceeding 45°). Our AR prototype based on the designed metasurface waveguide, exhibits superior color accuracy and uniformity. This unique achromatic metasurface waveguide technology is expected to advance the development of visually compelling experience in compact AR display systems.

## Introduction

Augmented reality, dating back to 1968^1^, continues to draw attention for its potential to revolutionize the ways we perceive and interact with the digital world. It brings new opportunities in miscellaneous cutting-edge fields such as metaverse, smart industry, smart healthcare, smart tourism, just to name a few^2–4^. Various optical architectures have been proposed for AR displays^5–10^, such as freeform mirrors^11^, birdbath^12^, Maxwellian view displays^13^, and waveguide combiners^10^. AR technology continuously advances toward ultracompact formfactor and lightweight designs, enhancing user experience. Diffractive waveguide combiners have emerged as the preferred option in commercial AR devices (Microsoft HoloLens^14^, Magic Leap^15^, Dispelix^16^, etc.) due to their eyeglass-like appearance, minimal weight, high throughput, and mass production capability. The waveguide solution utilizes a slim planar glass integrated with diffractive input and out-couplers. Image-bearing light rays enter the waveguide through the in-coupler, propagate via total internal reflection (TIR), and are extracted toward the user’s eyes by the out-coupler. From this display process, these couplers serve as the pivotal components determining the imaging quality.

Restricted by the spectral spread induced by diffraction, these diffractive couplers suffer from considerable chromatic dispersion and thus severely limits their full-color displays applications. To mitigate such an issue, a straightforward way is to use a symmetric in-coupler and out-coupler configuration. Although the spectral spread can be compensated by the configuration, the light of different wavelengths is spatially displaced at the out-coupler, which will induce a strong color nonuniformity over the eyebox. Additionally, spectral spread reduces the effective FOV for RGB light propagation through TIR^17,18^ (Supplementary Information). Consequently, a common approach to eliminate chromatic dispersion without FOV compromise involves utilizing three waveguides, each dedicated to a specific RGB color. However, this method inevitably increases the system complexity, causes ghost images and reduce the modulation transfer function (MTF) due to waveguide misalignment^10,17^. Moreover, conventional couplers face challenges in achieving illuminance uniformity across the entire RGB FOV, primarily due to the complexities of manipulating diffraction efficiency across both spectral and angular domains in the in-coupler and out-coupler^19^.

Metasurface, a new type of optical element, has been attracting huge attention and undergone rapid development in recent years^20–24^. Composed of nanostructures at a scale comparable to or smaller than the wavelength of light^6,8,9^, metasurface can manipulate light’s phase, amplitude, and polarization through resonant phase^25^, propagation phase^26^, geometric phase^27^ or nonlocal phase modulation^28^. The sophisticated light manipulation capabilities of metasurfaces are now being integrated into cutting-edge display technologies, including meta-OLED^29^, holography^30^, 3D displays^31^, and virtual reality^32^, making a significant advancement in optical engineering. Recent years have witnessed the great potential of metasurfaces in AR display, attributed to their high degree of design freedom and flexible light-field modulation capability^33–39^. Pioneering efforts in exploiting metasurface-based waveguide AR displays have achieved notable progress in broadening angular bandwidth^40^, improving efficiency^41^, enlarging the FOV^42^, and wavelengths multiplexing^43^. Regarding chromatic aberration correction in waveguide AR displays, while dispersion-compensating waveguide offer a solution, it result in limited FOV and fixed small exit pupil^44^. as Alternative approaches include metagratings constructed with nine-stacked dielectric layers of titanium dioxide (TiO_2_) and silicon dioxide (SiO_2_)^45^, and metastructures composed of three layers of aluminum (Al), silver (Ag), and gold (Au)^46^. It is worth mentioning that a majority of achromatic metasurface couplers exploited so far rely on intricate multi-layer nanostructures to enhance design flexibility and attain desired dispersion control. However, this approach incurs challenging manufacturing process. Furthermore, the strong angular spread inherent in the couplers inevitably leads to a degradation in both color and angular uniformity.

**Fig. 1:**
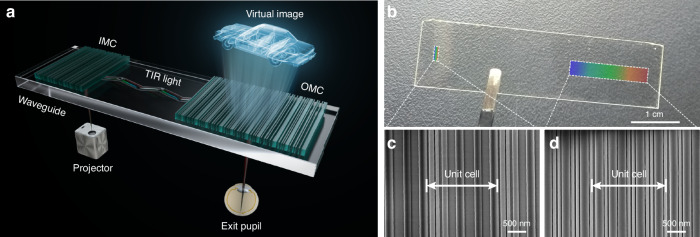
Schematic illustration of metasurface waveguide AR display system and fabricated sample. **a** AR system comprising metasurface couplers and high refractive index waveguide. The input metasurface coupler (IMC) couples the virtual image from the projector into the waveguide, where the optical beams propagate horizontally via total internal reflection (TIR) to the output metasurface coupler (OMC) positioned before the human eye, while allowing ambient light transmission through the waveguide. **b** Photograph of the fabricated metasurface waveguide with 1 mm thickness. Both IMC and OMC are fabricated using silicon nitride (SiN, *n* = 2.0). **c**, **d** Scanning electron microscope (SEM) images of the IMC and OMC, respectively

Here, we demonstrate a metasurface waveguide AR display that combines in- and output metasurface couplers and a high refractive index waveguide in a compact form factor, as shown in Fig. 1a. The experimental prototype based on our designed metasurface waveguide demonstrates high-quality full-color AR image display. Compared with conventional diffractive waveguides, our metasurface waveguide design featuring a single-layer structure enables significant device miniaturization and effectively suppresses waveguide dispersion. The waveguide system achieves superior color and angular uniformity that meets the stringent requirements of AR applications. This breakthrough enables full-color display without multi-guide alignment while ensuring optimal MTF at reduced manufacturing costs. Such exceptional performance is achieved through inverse-designed metasurface couplers based on adjoint method^47–50^. The method facilitates automated development of intricate structures with unique functionalities beyond classical metasurfaces. Our metasurface waveguide proposed here empowers full-color display with high uniformity and color accuracy in a single-layer manner, paving the way for user-friendly wearing experience of AR glasses.

## Results

Figure 1a illustrates the optical architecture of our metasurface waveguide AR display system. The system comprises an input metasurface coupler (IMC), an output metasurface coupler (OMC), and a single-plate waveguide. Both metasurface couplers are fabricated on the same side of the waveguide and operate in reflection mode. The IMC couples light from an external projector into the waveguide, where it propagates through TIR. The OMC provides horizontal eye-box extension and couples light to the observer’s pupil. Simultaneously, real-world scene light transmits through the waveguide and OMC to the observer, realizing the fusion of the virtual and real worlds. The k-vector of the metasurface couplers $({\overrightarrow{K}}_{IMC},{\overrightarrow{K}}_{OMC})$ satisfy a condition ${\overrightarrow{K}}_{IMC}+{\overrightarrow{K}}_{OMC}=0$, ensuring conservation of in-plane k-vector components between input and output light for distortion-free image transfer. Figure 1b shows the fabricated achromatic metasurface waveguide. Figures 1c and d present the top-view scanning electron microscope (SEM) images of the IMC and OMC, respectively. Both figures contain repeating stripe structures with varied widths.

**Fig. 2:**
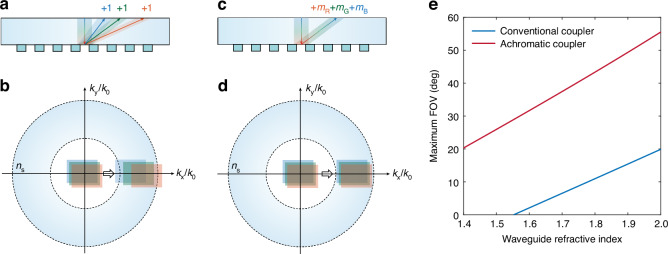
Comparison between conventional coupler and achromatic metasurface coupler. **a** Conventional coupler typically coupling light into the waveguide through first-order diffraction. **b** Normalized k-vector diagram of the conventional coupler. **c** Achromatic metasurface coupler coupling light into parallel beams within the waveguide through different high-order diffractions. **d** Normalized k-vector diagram of the achromatic metasurface coupler. **e** Calculated maximum FOV as a function of refractive index for both conventional and achromatic metasurface coupler waveguides (Supplementary Information)

We compare the optical response characteristics between conventional and achromatic metasurface couplers. As depicted in Fig. 2a, conventional couplers utilizing first-order diffraction exhibit wavelength-dependent deflection angles, resulting in visual color shift. In contrast, the ideal achromatic coupler shown in Fig. 2b deflects RGB-wavelength light to identical view angle. To design such a coupler, we start from basic principles and leverage the reflection mode grating equation,

1 $${n}_{s}\cdot sin{\theta }_{i}-{n}_{s}\cdot sin{\theta }_{m}=m\lambda /\varLambda$$

where ${\theta }_{i}$, ${\theta }_{m}$ are the incident and diffracted angles respectively, ${n}_{s}$ is the refractive index of the waveguide, $m$ is the diffraction order, $\lambda$ is the wavelength, and $\varLambda$ is the period of the metasurface coupler. It is clear that a diffractive element can realize achromatic diffraction if the following equation is satisfied.

2 $${m}_{R}{\lambda }_{R}={m}_{G}{\lambda }_{G}={m}_{B}{\lambda }_{B}$$

where subscripts RGB represent red, green, and blue colors. Appropriate selection of diffraction orders for RGB wavelengths enables color shift elimination, as illustrated in Fig. 2c. In traditional diffraction waveguides, chromatic aberration significantly impacts the FOV size. The dispersive nature of the in-coupler results in wavelength-dependent coupling angles, with longer wavelengths experiencing larger angles. This reduces the overlapping region of RGB FOV within the waveguide. In addition, the spectral spread of the in-coupler also stretches the RGB exit pupils too far apart, leading to different pupil replication densities and increased color nonuniformity in the eyebox^17^. It reveals the limitations of the conventional couplers used in a full-color AR display. Though this single-plate waveguide constraint can be overcome by stacking three RGB waveguides, it will make the optical combiner thicker and heavier.

Figure 2d shows reduced spectral spreading and smaller color shift through the achromatic metasurface coupler. The maximum FOV calculations of achromatic metasurface and conventional couplers (Supplementary Information) are presented in Fig. 2e. The metasurface coupler demonstrates superior performance with a 45° FOV, significantly exceeding that of conventional couplers. The calculations in Fig. 2e employ RGB wavelengths of 663 nm, 530 nm and 442 nm, respectively, as determined by Eq. (2). This conclusion remains valid across different wavelength selections.

In our design, the diffraction orders of 4th, 5th, and 6th are assigned to RGB wavelengths of 663 nm, 530 nm, and 442 nm, respectively. Specifically, the IMC utilizes +4th, +5th, and +6th diffraction orders to couple the signal light into the waveguide, while the OMC employs corresponding -4th, -5th, and -6th orders to extract light toward the observer’s eye. Both couplers share the same periodicity of 1900 nm. Notably, while the grating equation determines the diffraction directions, it does not specify the power distribution between desired and unwanted orders. Poor coupling efficiency uniformity remains a critical challenge for waveguide AR display, particularly for large incident angles near the edge of the FOV^40^.

Simultaneous precise control of optical response at multiple wavelengths (663 nm, 530 nm, 442 nm) and wide angular range (−22.5° ~ 22.5°) poses substantial challenges, particularly for high diffraction orders. We utilize a rigorous-coupled-wave-analysis solver^51^ to optimize the geometry of the metasurface couplers. Details of the algorithm are presented in the Supplementary Information. The metasurface couplers are placed on the same side of waveguide (SCHOOT, *n* = 1.9) and illuminated by RGB-wavelength light with transverse electrically (TE) polarization. The simulations concerning transverse magnetic (TM) polarization are presented in the Supplementary Information. The height of etched silicon nitride nanostructures (SiN, *n* = 2.0) are 160 nm for both IMC and OMC. The figure of merit $(FoM)$ to be minimized during the optimization is the combination of the various squared ‘distances’ ${f}_{n}$ between the target diffraction efficiency and calculated diffraction efficiency of a designed geometry, $FoM({f}_{n})$, ${f}_{n}={|{\eta }_{m}-{\eta }_{target}|}^{2}$.

**Fig. 3:**
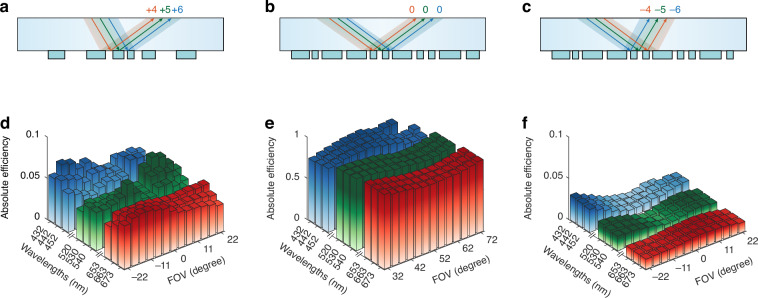
Working principles and simulations of the inverse-designed achromatic metasurface couplers. Illustration of RGB lights **a** couple into the input metasurface coupler through different diffraction orders, **b** travel inside waveguide in the exit pupil region (zeroth diffraction orders), **c** couple out from the output metasurface coupler through different diffraction orders. **d**–**f** Corresponding to the processes illustrated in **a**–**c** respectively, the absolute efficiency as a function of RGB wavelengths and FOV

Figure 3 shows the diffraction efficiency of inverse-designed IMC and OMC across the RGB spectral range and FOV. The IMC couples the RGB signal light into waveguide using 4th, 5th and 6th diffraction orders, as illustrated in Fig. 3a. Subsequently, Fig. 3b demonstrates horizontal eye-box extension through 0th diffraction order of the OMC, while Fig. 3c shows the OMC coupling signal light to the observer’s pupil using diffraction orders opposite to the IMC. The corresponding absolute efficiency as a function of the RGB wavelengths and FOV are presented in Fig. 3d–f. The IMC achieves an average efficiency of approximately 5%, while the OMC demonstrates average efficiencies of 80% for 0th-order diffraction and 2% for high-order diffraction. Quantitative analysis of the metasurface waveguide uniformity across color and angular ranges reveals high uniformities of 72.8%, 85.1%, and 46.3% for the processes illustrated in Fig. 3a, b, and c, respectively. The following definition of uniformity $U$ is adopted:

3 $$U=1-\frac{{\eta }_{max}-{\eta }_{min}}{{\eta }_{max}+{\eta }_{min}}$$

where ${\eta }_{min}$ and ${\eta }_{max}$ represent the minimum and maximum efficiency through the color and angular ranges, respectively. Notably, these full-color waveguide results approach the theoretical values reported for monochromatic waveguides^19^. These findings confirm that our inverse-designed metasurface couplers achieve highly consistent coupling efficiency at target diffraction orders during full-color operation. The efficiency remains stable even with 20 nm RGB spectral linewidth.

**Fig. 4:**
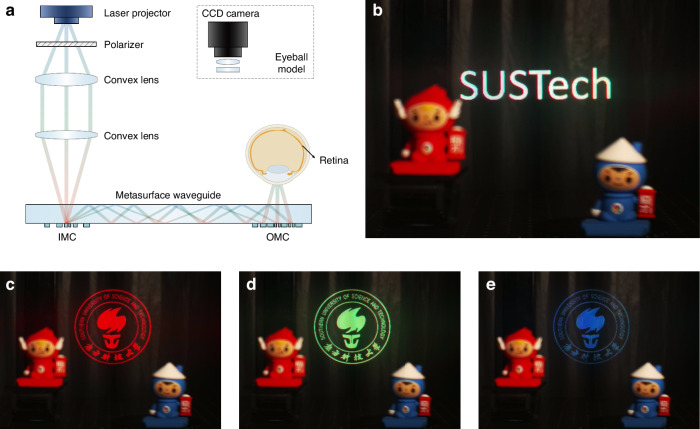
The AR display prototype and demonstration results using the metasurface waveguide display. **a** Schematic of the AR near-eye display system comprising an RGB-achromatic metasurface waveguide and a laser projector. A CCD camera simulates the eyeball model. **b** The AR displayed image, showing white “SUSTech” letters superimposed on a real-world scene containing two dolls. **c**–**e** AR-displayed SUSTech logos in red, green, and blue colors, respectively. All images were captured via the CCD camera

Figure 4a presents our AR display prototype, comprising the fabricated metasurface waveguide and a laser projector. The beam from the laser projector is first passing through a TE-polarizer and then refocused by two convex lenes. The image is projected onto the retina via the metasurface waveguide to create a virtual scene. In our experiment, we used a CCD camera to mimic the eyeball model. The center emission wavelengths of the projector are 639 nm (red), 522 nm (green), and 445 nm (blue), which are slightly deviate from the designed wavelengths. While this deviation maintains image integrity due to in-plane momentum conservation between input and output light. But as previously stated, it does stretch the RGB exits pupils apart and affects color balance within the eyebox.

**Fig. 5:**
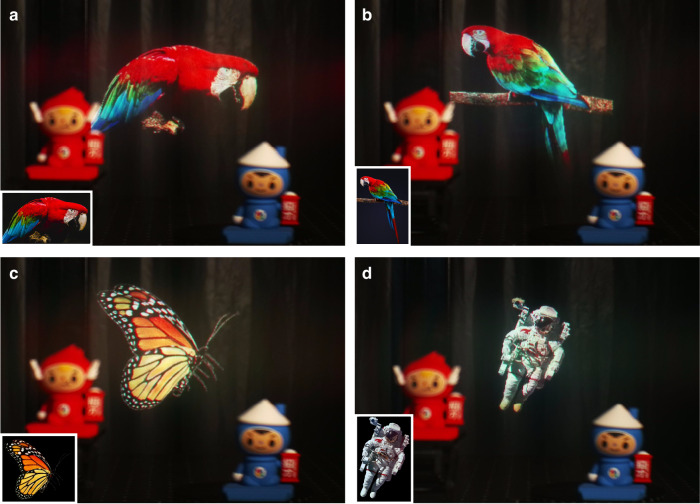
Experimental results captured through our metasurface waveguide AR display prototype. **a**–**d** Images captured via a CCD camera in the optical-see-through AR mode. The parrots, butterfly, astronaut are digitally superimposed objects, while two dolls are real see-through objects. Insets show the original projector images. Across all examples, the proposed metasurface waveguide faithfully reproduces the digital objects with high color uniformity and fidelity

Figure 4b demonstrates the floating virtual image of white letters captured from our metasurface waveguide prototype, showing clear character display with accurate RGB color mixing. The individual RGB illumination results of the SUSTech logo in Fig. 4c–e verify precise single-color image formation. To validate real-world application capabilities, Fig. 5 presents augmented reality images captured by the CCD camera, featuring test images with complex color compositions such as parrot, butterfly, and astronaut. All captured virtual images demonstrate vivid reproduction of input images. The high fidelity between virtual and original images further confirms the AR performance of our metasurface waveguide. A demonstration video is provided in the supplementary information (Supplementary Movie S1).

## Discussion

The proposed achromatic metasurface waveguide uses ultra-thin metasurface (full binary SiN nanostructures) couplers and single-plate waveguide to enable simple and cost-effective fabrication for full-color AR displays. The metasurface couplers effectively take advantage of different diffraction orders for RGB wavelengths in reflection mode, achieving high color and angular uniformity through optical response optimization. Our design provides a viable solution to the longstanding chromatic aberration issue in diffractive waveguide AR displays. It should be noted that the limitations imposed by the wavelength mismatch between the utilized and designed RGB light, along with inherent prototype aberrations have constrained the full potential of our design.

Improving the efficiency of our achromatic metasurface waveguide AR display requires optimization of the input coupler, potentially through the strategic application of reflective coatings^17^. Given the polarization selectivity inherent in our system, future endeavors will focus on the design and implementation of 2D polarization-insensitive metasurfaces, thereby broadening the applicability of our waveguide design. Additionally, as detailed in the Supplementary Information, suppressing excessive high-order diffraction is crucial for the realization of wide-field displays. Alternative approaches to achromatic designs devoid of higher-order diffraction may benefit from multi-period gratings^52^ or extraordinary optical diffraction^30^, offering promising pathways for advanced AR display development.

Finally, our achromatic metasurface waveguide technology demonstrates unique advantages in full-color display, large supported FOV, compact form factor, and low manufacturing costs, showing significant promise for future AR display applications.

## Methods

### Simulations

Numerical simulations of electromagnetic field are implemented with MATLAB library (Reticolo) based on RCWA. The diffraction efficiency is calculated through RCWA simulations and the calculated gradient value is updated based on hyperparameters of the Adam optimizer. The optimization simulation is carried out on a workstation with Intel Gold 5218R CPU and all iterative calculations are parallel. The polarization of incident light is utilized as TE-polarized light changing the elevation angle. All results are numerically verified using the commercial FDTD software (Ansys Lumerical).

### Fabrication

Firstly, a 160-nm-thick silicon nitride film is deposited on the high-refractive index waveguide at the temperature of 300 °C using plasma enhanced chemical vapor deposition (PECVD) (Oxford Plasma Pro 100 Cobra 180) with a gas mixture of SiH_4_, NH_3_ and N_2_. After removing the residual organics on the film, the positive electron-beam resist (ZEP520) is spin-coating on the silicon nitride film. The metasurface couplers patterns are then defined in this high-resolution positive resist by an electron-beam lithography (EBL) system (JEOL9500) at 100 KV. Next, the reactive-ion-etch (RIE) (Oxford Instrument Plasmalab System 100 RIE180) with a mixture of CHF_3_, SF_6_, and N_2_ gases is applied to etch through the 160-nm-thick silicon nitride layer. The final sample can be obtained after removal of the residual resist by the dry etching with the O_2_ plasma descum.

## Supplementary information

Supplementary Information for Achromatic metasurface waveguide for augmented 1 reality displays

Supplementary Video 1

Supplementary Video 2

## Data Availability

All data needed to evaluate the conclusions in the paper are present in the paper. Additional data related to this paper may be requested from the authors.
