# Supplementary material for: An achromatic metasurface waveguide for augmented reality displays
Source: Light Sci Appl. 2025 Feb 25;14:94. doi: 10.1038/s41377-025-01761-w (PMC11850613; doi:10.1038/s41377-025-01761-w)
Supplement: Supplementary file 1 — Supplementary Information for Achromatic metasurface waveguide for augmented 1 reality displays [file 41377_2025_1761_MOESM1_ESM.docx]

# Supplementary Information for

**Achromatic metasurface waveguide for augmented reality displays**

# Zhongtao Tian^1,2^, Xiuling Zhu^1^, Philip A. Surman^1^, Zhidong Chen^2^, Xiao Wei Sun^1, *^

*^1^Institute of Nanoscience and Applications, and Department of Electrical and Electronic Engineering, Southern University of Science and Technology, Shenzhen 518055, China.*

*^2^PengCheng Laboratory, Shenzhen 518055, China.*

*Corresponding author: [sunxw@sustech.edu.cn](mailto:sunxw@sustech.edu.cn)

# This PDF file includes:

Section S1. Maximum FOV as a function of the refractive index of the waveguide

Section S2. Inverse-design of metasurface couplers

Section S3. Ghost images

Section S4. The combination of wavelengths and diffraction orders to achieve achromatic metasurface couplers

Fig. S1. Schematic illustration of input metasurface coupler with a waveguide.

Fig. S2. Ghost images test setup and results.

Fig. S3. Optimization process of the inverse-designed metasurface couplers.

Fig. S4. Simulations for the metasurface couplers using TM polarization light.

Fig. S5. Simulations for the metasurface couplers with center emission wavelengths of 639 nm (red), 522 nm (green), and 445 nm (blue).

Fig. S6. Top view of the metasurface waveguide.

Fig. S7. Captured images of AR display under daylight conditions.

Fig. S8. Captured images of AR display after adjusting the exposure time of the CCD camera.

Fig. S9. Captured real-scene images (left) without or (right) with the metasurface waveguide.

Fig. S10. Captured images of the fabricated metasurface waveguide.

Fig. S11. A qualitative explanation of the chromatic aberration correction capability of our metasurface waveguide.

Movie S1. An AR imaging movie.

Movie S2. Metasurface couplers topology optimization animation.

**Section S1. Maximum FOV as a function of the refractive index of the waveguide**


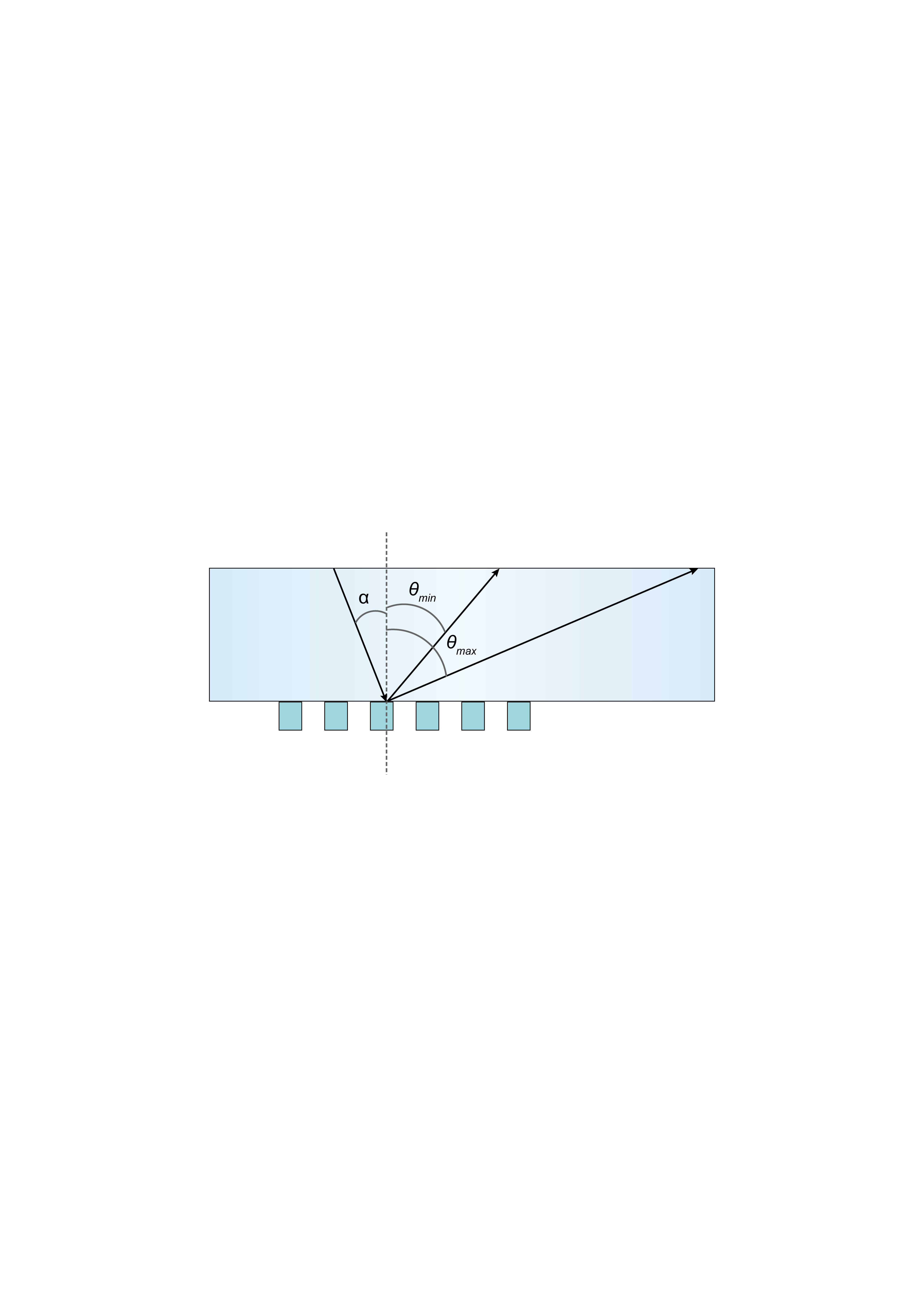


Figure S1. Schematic illustration of input metasurface coupler with a waveguide.

The waveguide with a refractive index (*n_wg_*) of 1.9 is utilized. And the refractive index of air is 1. According to the grating equation, the output diffraction angle (*θ*) is determined as follows:

$$n_{wg}\sin\theta-n_{wg}\sin\alpha=\frac{m_{i}\lambda_{i}}{\Lambda}$$

Where *α* is the input angle, *m_i_* is the *mth* diffraction order, λ_i_ is the target wavelength and Λ is the period of the metasurface coupler.

For the diffracted light to propagate along the waveguide via total internal reflection (TIR), the diffraction angle *θ* must fall in the range of *θ_min_* ≤ *θ* < *θ_max_*. It corresponds to the edges of field of view (FOV). In this paper, *θ_min_*=*θ_c_* = *arcsin*(1/*n_wg_*) is the total reflection angle and *θ_max_*=75° to prevent gaps between each two TIRs. This condition holds for all the RGB-wavelength light. Then we can obtain:

$$\left\{\begin{matrix} n_{wg}\sin(-\frac{FOV_{wg}}{2})+\frac{m_{i}\lambda_{i}}{\Lambda}>n_{wg}\sin\theta_{\min} \\ n_{wg}\sin(\frac{FOV_{wg}}{2})+\frac{m_{j}\lambda_{j}}{\Lambda}<n_{wg}\sin\theta_{\max} \end{matrix}\right.$$

Further, it can be deduced that:

$$\left\{\begin{matrix} \Lambda<\frac{m_{i}\lambda_{i}}{n_{wg}\sin\theta_{\min}-n_{wg}\sin(-\frac{FOV_{wg}}{2})} \\ \Lambda>\frac{m_{j}\lambda_{j}}{n_{wg}\sin\theta_{\max}-n_{wg}\sin\frac{FOV_{wg}}{2}} \end{matrix}\right.$$

To ensure that the Λ exists,

$$\frac{m_{i}\lambda_{i}}{n_{wg}\sin\theta_{\min}-n_{wg}\sin(-\frac{FOV_{wg}}{2})}>\frac{m_{j}\lambda_{j}}{n_{wg}\sin\theta_{\max}-n_{wg}\sin\frac{FOV_{wg}}{2}}$$

For conventional coupler utilizing first-order diffraction, the FOV is determined by both RB wavelengths and waveguide refractive index.

$$\begin{matrix} FOV_{wg}<2\arcsin(\frac{\lambda_{B}\sin\theta_{\max}-\lambda_{R}\sin\theta_{\min}}{\lambda_{B}\text{+}\lambda_{R}}) \\ FOV_{air}<2\arcsin(n_{wg}(\frac{\lambda_{B}\sin\theta_{\max}-\lambda_{R}\sin\theta_{\min}}{\lambda_{B}\text{+}\lambda_{R}})) \end{matrix}$$

For achromatic metasurface coupler proposed in this work, the FOV depends solely on the waveguide refractive index due to *m_R_*λ_R_ = *m_G_*λ_G_ = *m_B_*λ_B_.

$$\begin{matrix} FOV_{wg}<2\arcsin(\frac{\sin\theta_{\max}-\sin\theta_{\min}}{2}) \\ FOV_{air}<2\arcsin(n_{wg}(\frac{\sin\theta_{\max}-\sin\theta_{\min}}{2})) \end{matrix}$$

**The inequality (3) establishes the permissible range for the grating period. Here, with a designed *FOV_air_* of 45**°, the corresponding periodicity interval spans from 1830 nm to 1910 nm. A period of Λ = 1900 nm is selected for this work.

**Section S2. Inverse-design of metasurface couplers**

We adopt a home-built topology optimization program to design metasurface couplers using the adjoint method. The adjoint method has gained attention in photonic device designs, including the design of metalenses^1,2^, metagratings^3–5^, and many other applications^6,7^, owing to its efficient gradient computation capability under high-dimensional degrees of freedom. Topology optimization based on the adjoint method enables photonic devices to have complex geometries with enhanced functionalities. We formulate the design problem as a minimization optimization of N distinct objective functions subject to Maxwell’s equations:

where *n*, *m* and *k* are positive integers, *f_n_* is each objective function dependent on the electric field ***E***, *FoM* is a single total objective function composed of these *f_n_*, ***ε*** is the relative permittivity as a function of the density design variables ***ρ*** at each point in space, ***J*** is the current density, and *g_k_* is the *k-*th constraint function.

We began with a random meta-design and underwent several optimization iterations. This process involved a forward simulation and an adjoint simulation to calculate the gradients of *FoM* until the device performance met the design criteria^3,4,6,8^. The equation below represents the gradients of *f_n_*, which will be combined to form the gradient of *FoM* after processing.

$$\frac{\partial f_{n}}{\partial\varepsilon}\propto\mathrm{Re}\left(t_{n}^{*}\mathbf{E}_{n}^{forward}\cdot\mathbf{E}_{n}^{adjoint}\right)$$

In order to parameterize the permittivity ***ε***, we first use a density-based filter and we choose a conic filter for the weights:

 (9)

Then, the resultant field is projected onto a binary value $\overline{\rho_{i}}$ using a differentiable and nonlinear function^9^.

$\overline{\rho_{i}}=\frac{\tanh(\beta\eta)+\tanh(\beta(\widetilde{\rho_{i}}-\eta))}{\tanh(\beta\eta)+\tanh(\beta(1-\eta))}$ (10)

The hyperparameters *β* and *η* control the binarization intensity and the threshold value, respectively. We initially set *η*=0.5 and *β*=3.0 and update *β* during the optimization processes. The combination of the density-based filter and the projection function can remove tiny features.

The final permittivity is then interpolated using:

$\mathbf{\varepsilon}_{\mathbf{r}}(\overline{\mathbf{\rho}})=\mathbf{\varepsilon}_{\min}+\overline{\mathbf{\rho}}(\mathbf{\varepsilon}_{\max}-\mathbf{\varepsilon}_{\min})$ (11)

where ***ε****_min_* is the permittivity of the "void" region (Air) and ***ε****_max_* is the permittivity of the "solid" region (SiN).

To ensure manufacturing feasibility, device geometries must satisfy the minimum feature size *d* achievable by the fabrication process. Without geometry constraints during optimization, final designs typically exhibit small features that are difficult to fabricate. To ensure fabrication requirements, we implement two geometric constraints that enforce a minimum length scale on both the solid and void regions of a topology^10^. These constraints are incorporated into the objective function as penalty terms after specified iterations. The minimum linewidth set here is 64 nm, compatible with nanoimprint lithography for mass production^11^.

The optimization process uses the gradient descent method, starting from a randomly initialized geometry and utilizing the gradient-based optimizer Adam^12^ to refine the profiles of the metasurface couplers. All simulations are performed in parallel to reduce the computation time.

**Section S3. Ghost images**


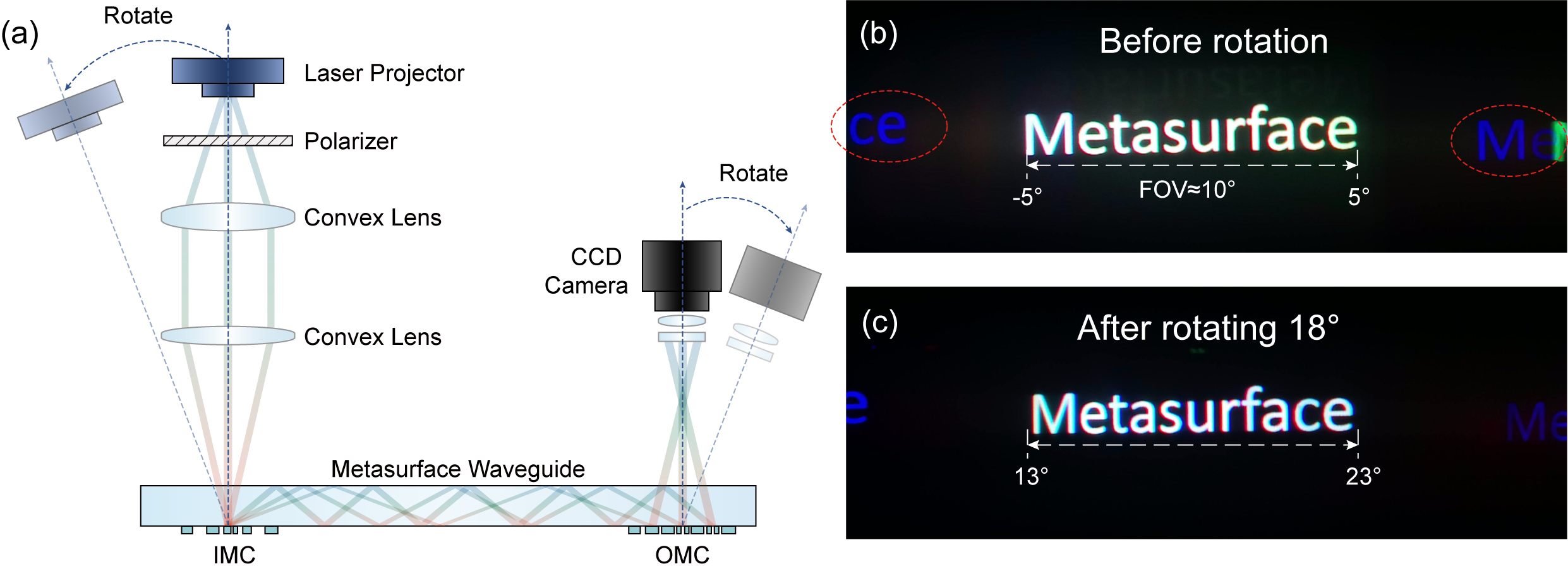


Figure S2. Ghost images test setup and results. **a** Schematic of ghost images test setup with rotatable projector and camera. **b** Imaging result of white letters before rotation. **c** Imaging result of white letters after rotating 18°.

Despite theoretical FOV exceeds 45° in the metasurface waveguide, ghost images superimposed on the normal image deteriorate display quality. To precisely characterize chromatic aberration correction within a 45-degree FOV, peripheral field imaging results are measured through synchronized rotation of projector and camera, as shown in Fig. S2 (a). Figure S2 (b) presents white letters imaging results at FOV of 10° before rotation, with incidence angles ranges from -5° to 5°. However, ghost images appear adjacent to the primary image (red dotted line). Following 18° rotation of projector and camera (Fig. S2(c)), with corresponding incidence angles from 13° to 23°. Similarly, residual ghost images persist.

These ghost images arise from the associated high-order diffraction. Large-period metasurface grating units are employed to generate the necessary high-order diffractions (e.g., ±4, ±5, ±6), with optimization focusing solely on desired diffraction orders. However, unconstrained redundant diffraction orders allow energy leakage into unutilized channels. Moreover, these high-order diffractions can propagate within the waveguide across specific angular ranges, contributing to ghost image formation.

In addition, the minimal color cast in captured images validates the RGB-achromatic characteristics of the designed metasurface waveguide across large FOV. From an implementation perspective, controlling redundant diffraction order energy remains crucial.

**Section S4. The combination of wavelengths and diffraction orders to achieve achromatic metasurface couplers**

The selection of diffraction orders and wavelengths is governed by the achromatic principle, which in this context can be represented as mλ=constant, where m represents the diffraction order and λ signifies the wavelength. This equation ensures that the chosen orders and wavelengths can be effectively coupled into the waveguide at the same angles. In theory, there can be various combinations to meet this condition (e.g. 720×3 = 540×4 = 432×6, 660×5 = 550×6 = 470×7 or any other similar combinations).

In our study, we opted for the 4th, 5th, and 6th diffraction orders coupled with wavelengths of 663 nm, 530 nm, and 442 nm, respectively. This particular combination was strategic for several reasons: the chosen wavelengths fall within the visible light spectrum, and the wavelengths were selected to be close to those emitted by the micro-projector (639 nm, 522 nm, 445 nm), ensuring compatibility and optimal utilization of the light source. Moderate orders help in avoiding the generation of unwanted diffraction orders that could lead to ghost images or reduce the overall system efficiency.


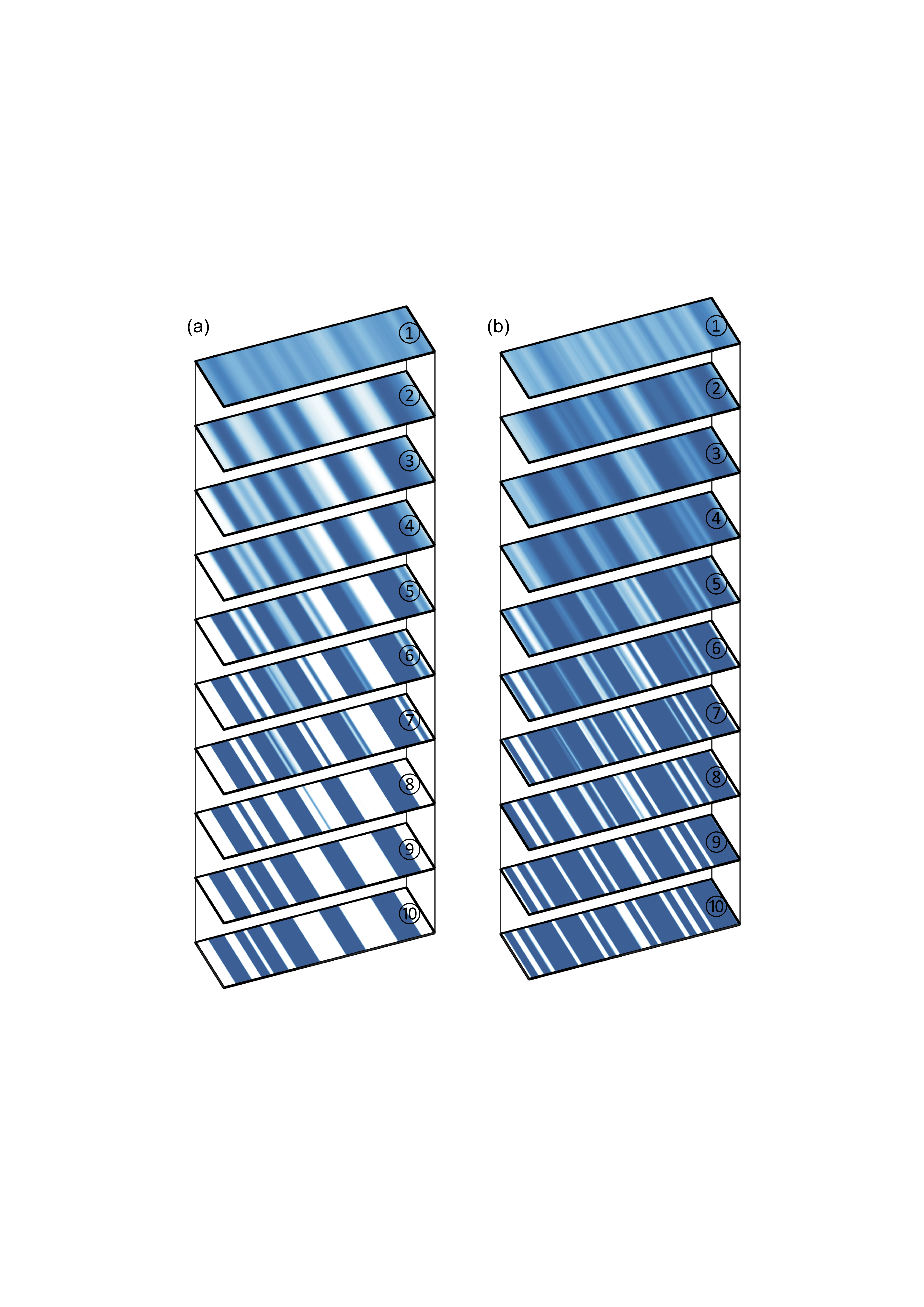


Figure S3. Optimization process of the inverse-designed metasurface couplers. (a) Geometry of the input metasurface couplers in the *xy* plane at the initial to final state (top to bottom). (b) Geometry of the output metasurface couplers in the *xy* plane at the initial to final state (top to bottom). The colour bar represents the refractive index at a wavelength of 530 nm.


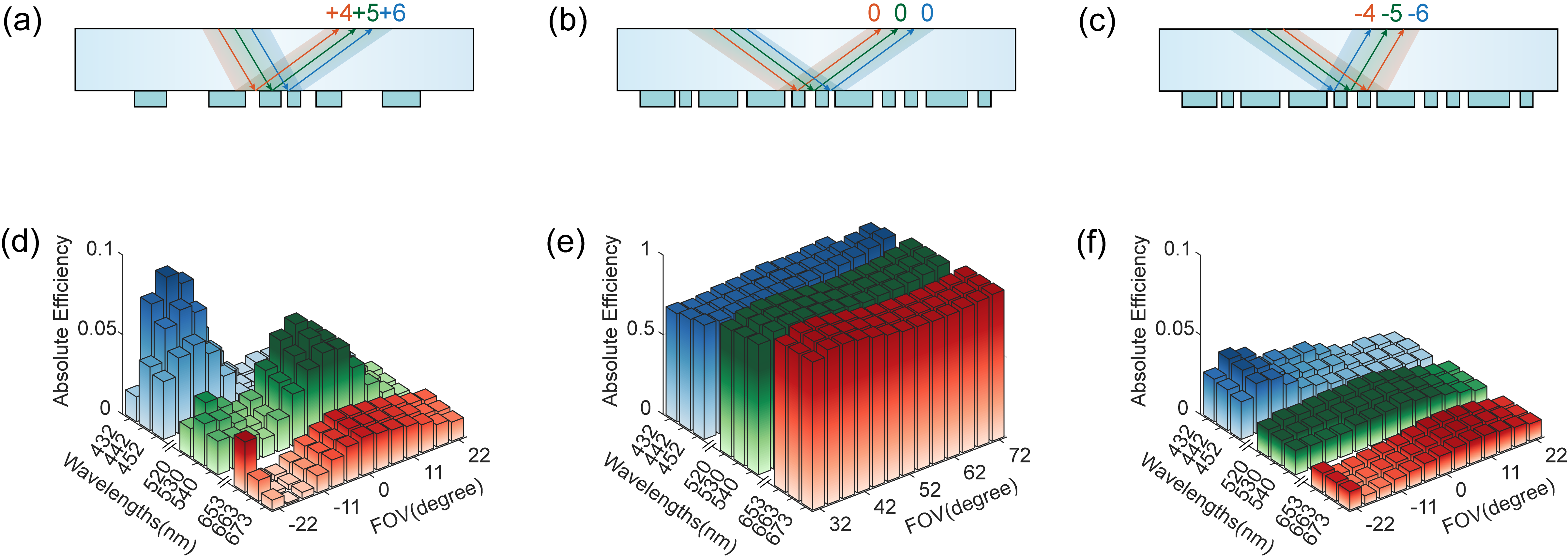


Figure S4. Simulations for the metasurface couplers using TM (Transverse Magnetic) polarization light.


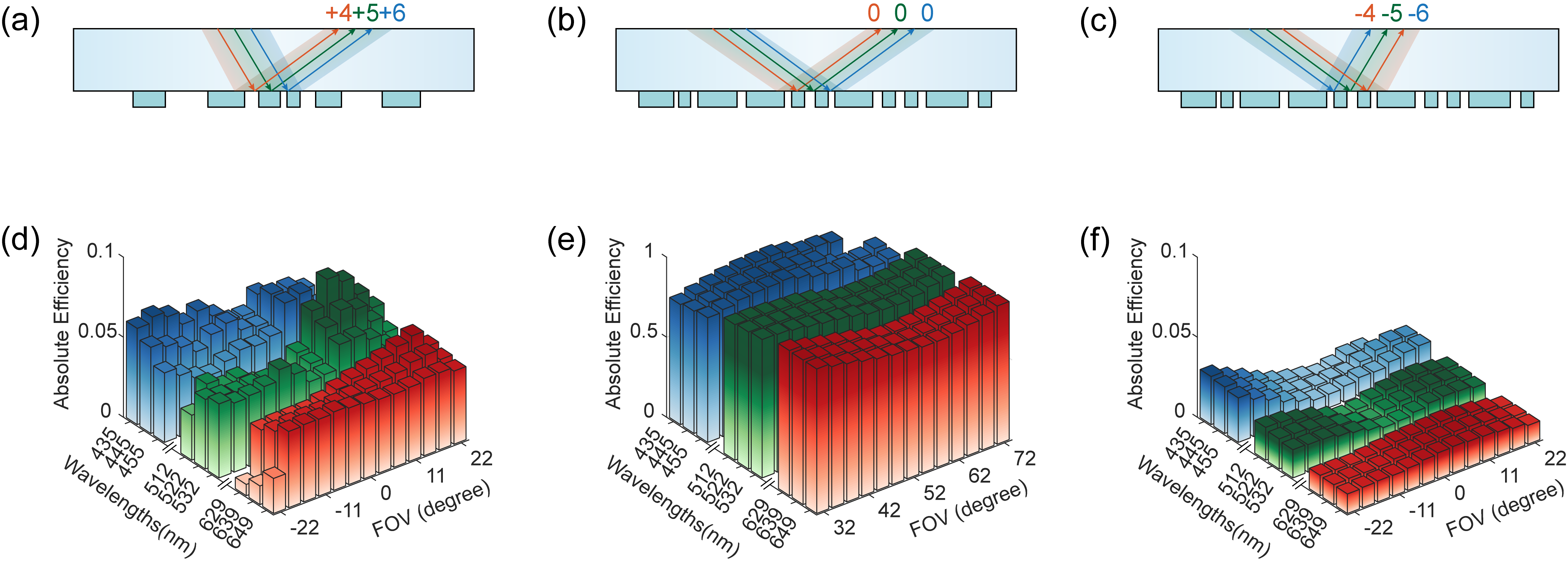


Figure S5. Simulations for the metasurface couplers with center emission wavelengths of 639 nm (red), 522 nm (green), and 445 nm (blue).


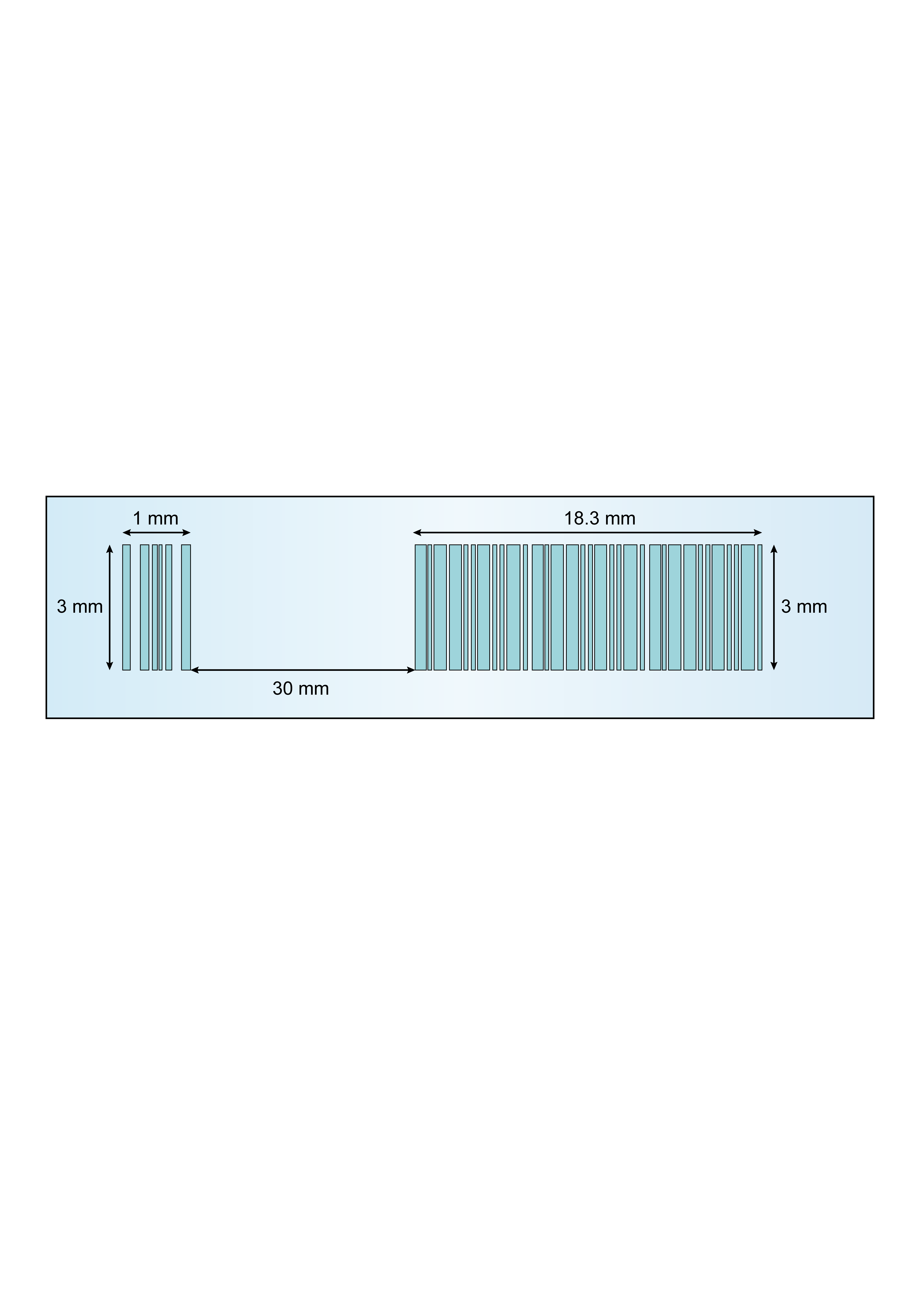


Figure S6. Top view of the metasurface waveguide, illustrating key distances within the structure. The image includes the positions and dimensions of the metasurface in- and out-couplers on the glass substrate.


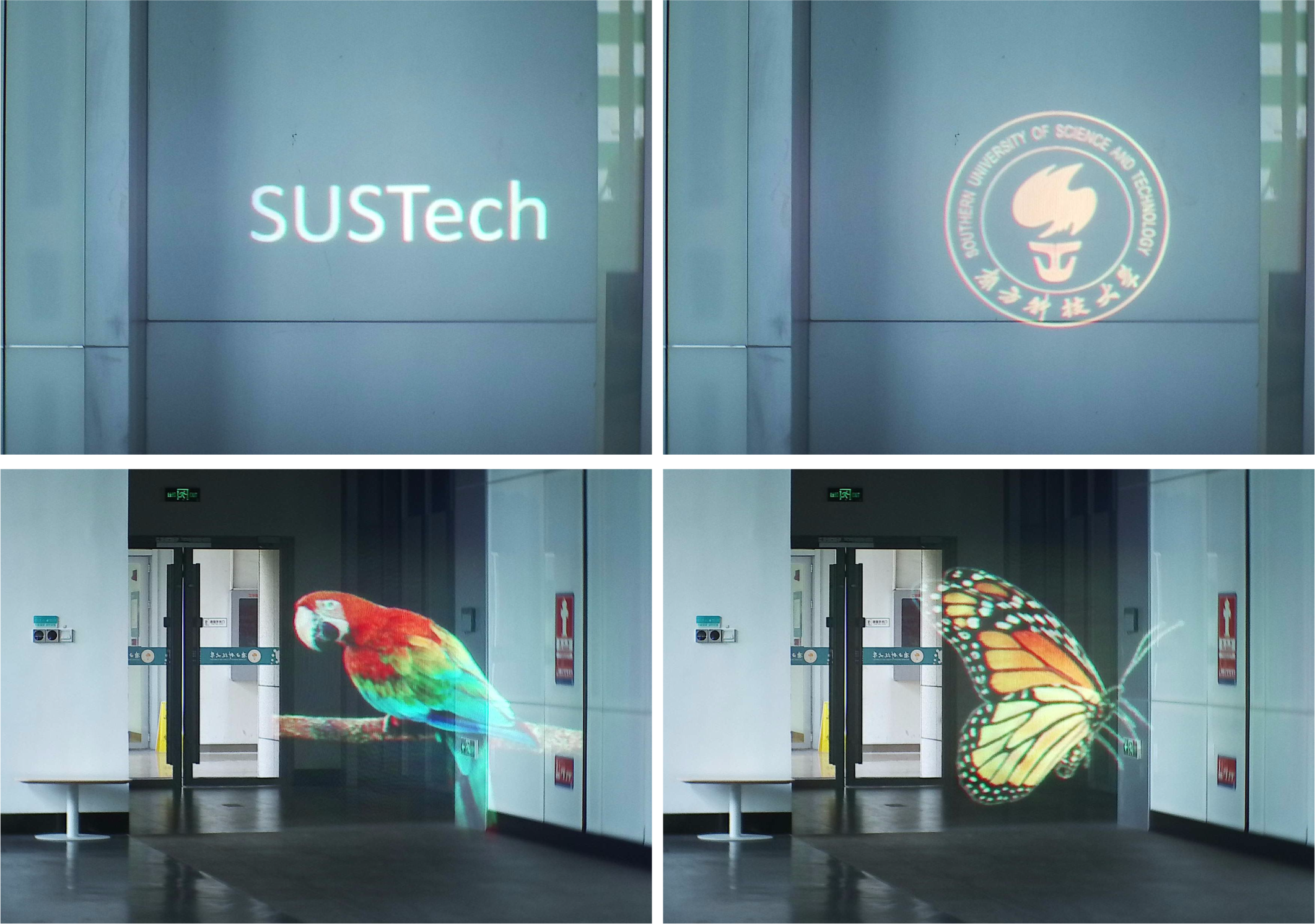


Figure S7. Captured images of AR display under daylight conditions.


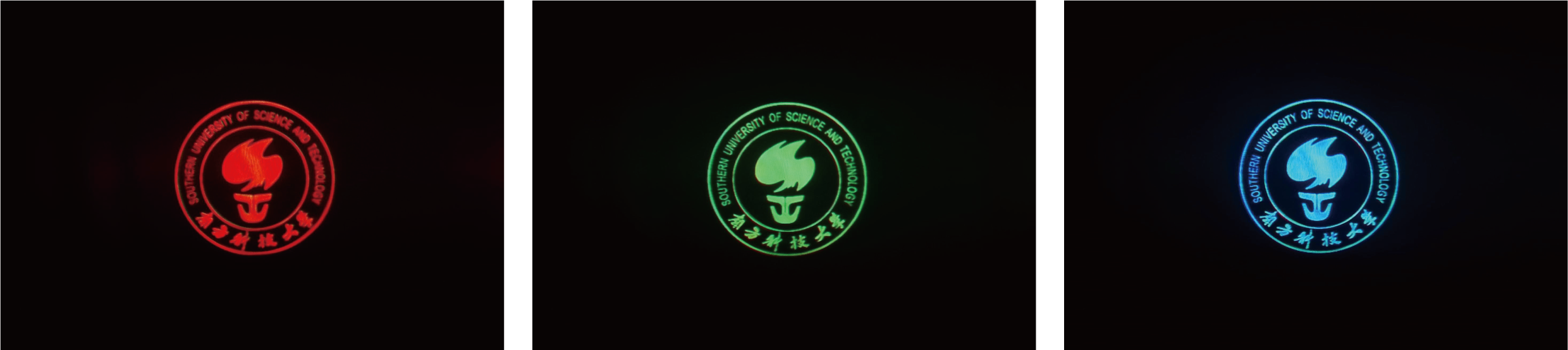


Figure S8. Captured images of AR display after adjusting the exposure time of the CCD camera. A more consistent display performance across the color spectrum is achieved. To attain better white balance and visual clarity, a joint optimization of the optical engine and the waveguide is imperative. This ensures uniform and clear display performance across all colors, providing superior visual experience.


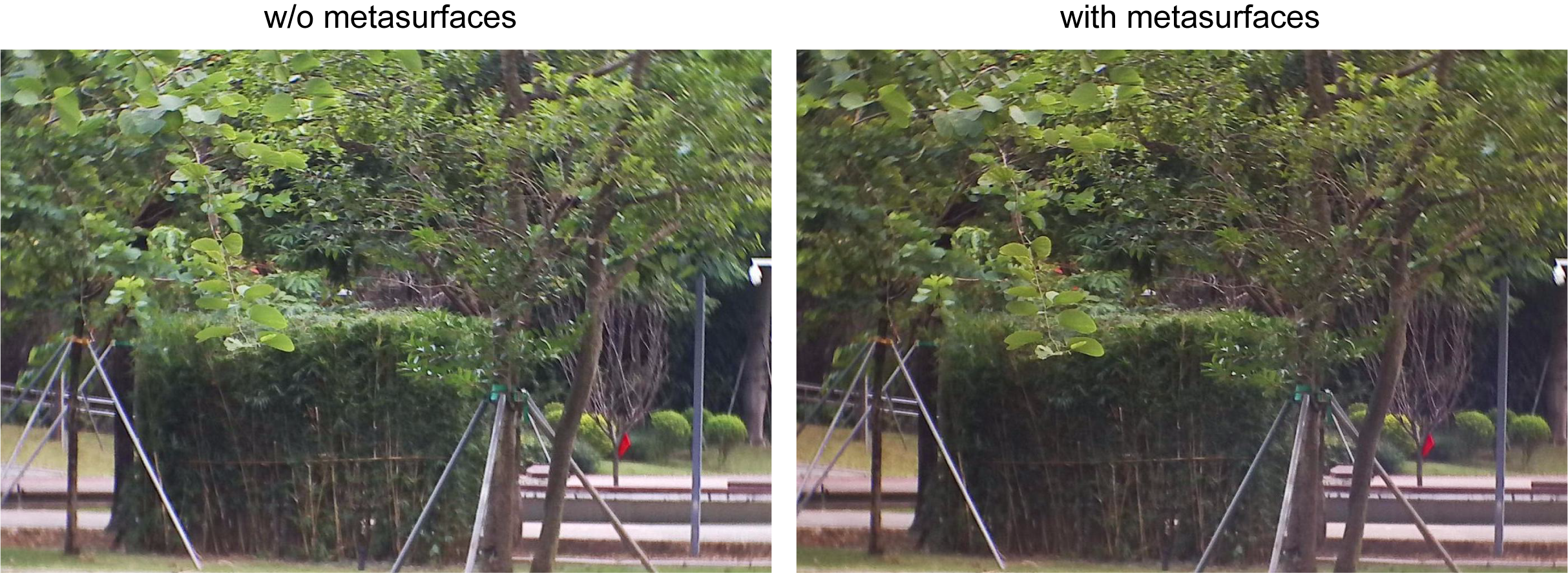


Figure S9. Captured real-scene images (left) without or (right) with the metasurface waveguide. The images were taken outside during the day using a CCD camera. Our inverse-designed metasurface waveguide provides high image clarity with significantly suppressed rainbow effect.


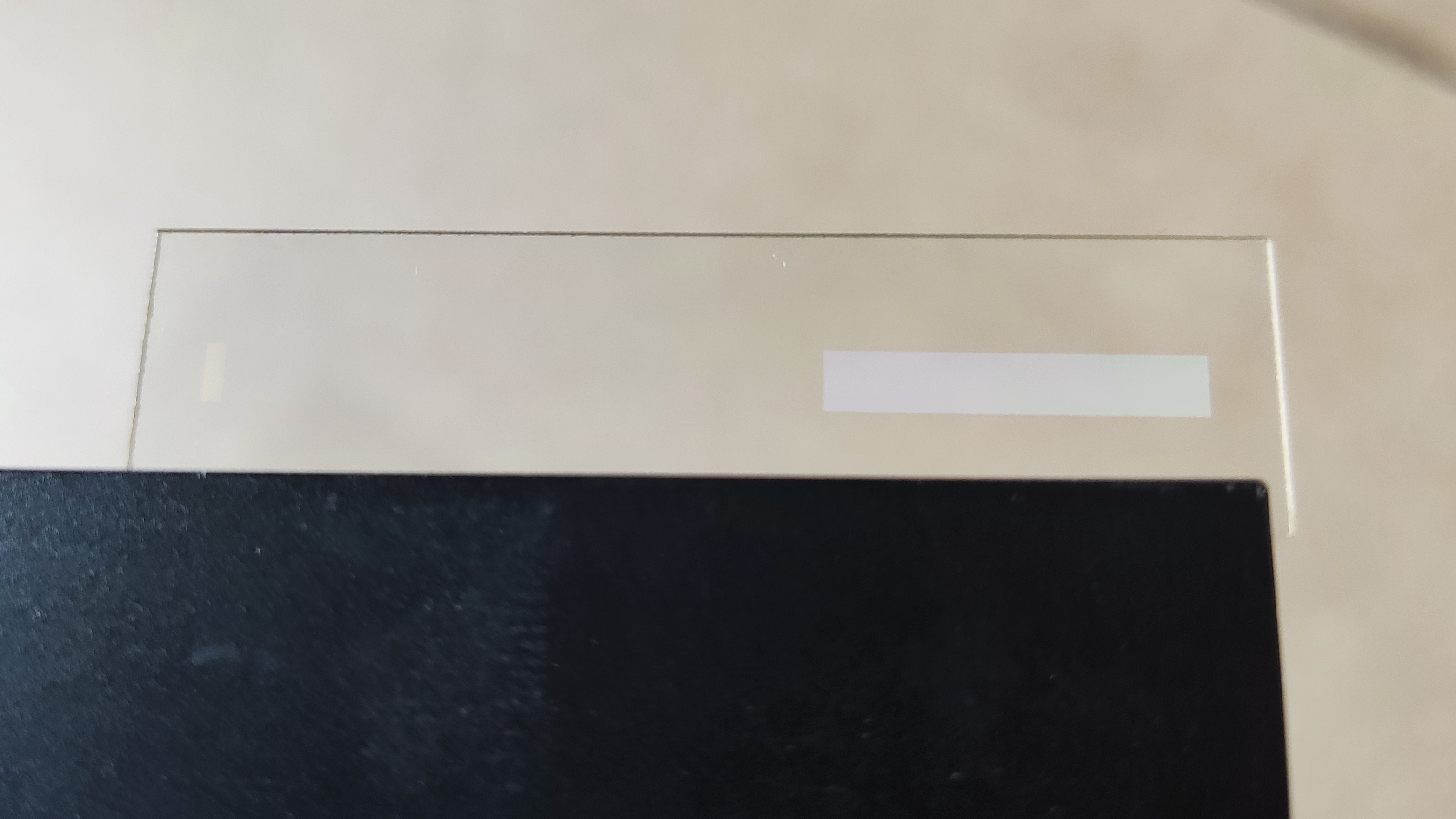


Figure S10. Captured images of the fabricated metasurface waveguide. Despite employing a high refractive index waveguide (n = 1.9) and achromatic metasurface design, the rainbow effect cannot be entirely mitigated. Distinct rainbow-like fringes remain observable at specific light source positions and viewing angles. Various strategies have been explored to address the rainbow effect in diffraction gratings for optical see-through AR displays, such as nonlocal metasurfaces^13^ and switchable polarization volume gratings^14^. These approaches represent a multifaceted effort to optimize the performance of diffraction gratings in AR displays while maintaining visual clarity and minimizing unwanted rainbow effect.


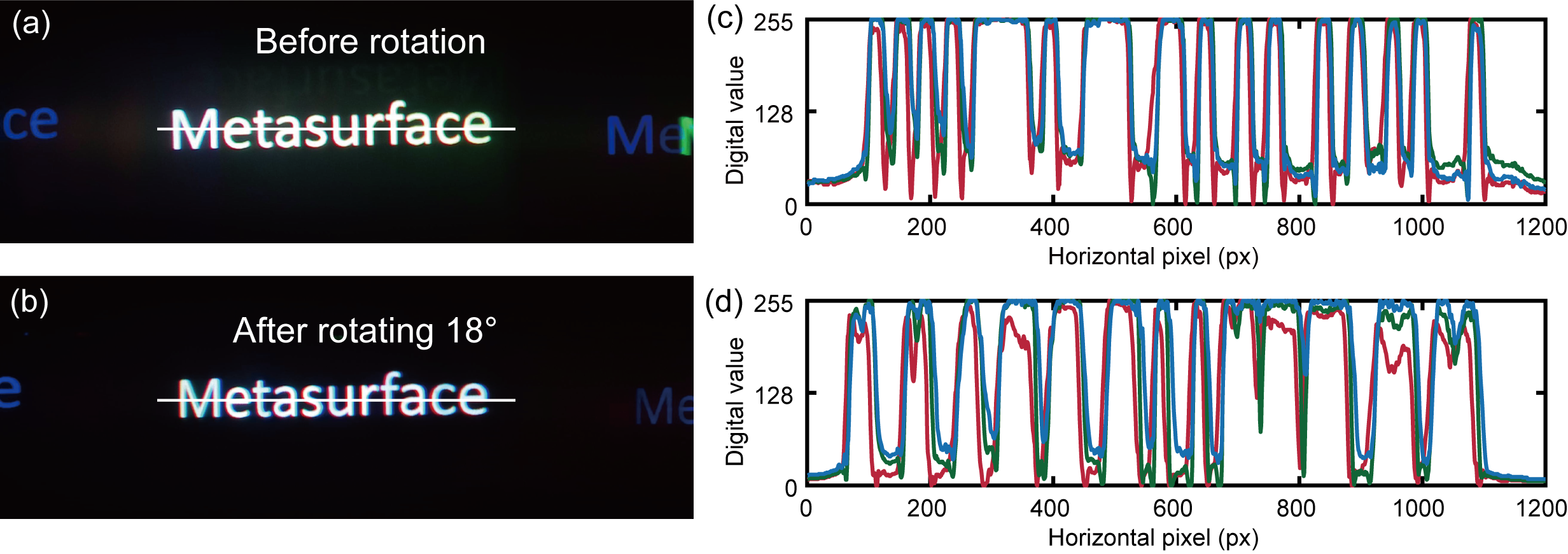


Figure S11. **a** Imaging result of white letters before rotation. **b** Imaging result following an 18° rotation. **c-d** The RGB pixel values extracted from regions marked by white lines in **a** and **b**, where line alignment indicates chromatic aberration correction capability. The observed near-perfect overlap confirms the high efficiency of our metasurface waveguide in mitigating chromatic aberration. The minor discrepancy noted in **d**, specifically the lower R line, is attributed to reduced red light efficiency at the peripheral field of view, which also explains the subtle blue cast observed in **b**. Meanwhile, some discrepancies in the alignment of the RGB lines, particularly in the lower sections of **c** and **d**, are attributed to noise interference.

**Movie S1.**

An AR imaging movie (30 fps).

**Movie S2.**

Metasurface couplers topology optimization process.

**References**

1. Phan, T. *et al.* High-efficiency, large-area, topology-optimized metasurfaces. *Light Sci. Appl.* **8**, 48 (2019).

2. Chung, H. & Miller, O. D. High-NA achromatic metalenses by inverse design. *Opt. Express* **28**, 6945–6965 (2020).

3. Sell, D., Yang, J., Doshay, S. & Fan, J. A. Periodic Dielectric Metasurfaces with High-Efficiency, Multiwavelength Functionalities. *Adv. Opt. Mater.* **5**, 1700645 (2017).

4. Sell, D., Yang, J., Doshay, S., Yang, R. & Fan, J. A. Large-Angle, Multifunctional Metagratings Based on Freeform Multimode Geometries. *Nano Lett.* **17**, 3752–3757 (2017).

5. Sell, D. *et al.* Ultra-High-Efficiency Anomalous Refraction with Dielectric Metasurfaces. *ACS Photonics* **5**, 2402–2407 (2018).

6. Molesky, S. *et al.* Inverse design in nanophotonics. *Nat. Photonics* **12**, 659–670 (2018).

7. Cordaro, A. *et al.* Solving integral equations in free space with inverse-designed ultrathin optical metagratings. *Nat. Nanotechnol.* **18**, 365–372 (2023).

8. Kim, D. C., Hermerschmidt, A., Dyachenko, P. & Scharf, T. Inverse design and demonstration of high-performance wide-angle diffractive optical elements. *Opt. Express* **28**, 22321–22333 (2020).

9. Wang, F., Lazarov, B. S. & Sigmund, O. On projection methods, convergence and robust formulations in topology optimization. *Struct. Multidiscip. Optim.* **43**, 767–784 (2011).

10. Zhou, M., Lazarov, B. S., Wang, F. & Sigmund, O. Minimum length scale in topology optimization by geometric constraints. *Comput. Methods Appl. Mech. Eng.* **293**, 266–282 (2015).

11. Einck, V. J. *et al.* Scalable Nanoimprint Lithography Process for Manufacturing Visible Metasurfaces Composed of High Aspect Ratio TiO2 Meta-Atoms. *Acs Photonics* **8**, 2400–2409 (2021).

12. Kingma, D. P. & Ba, J. Adam: A Method for Stochastic Optimization. Preprint at https://doi.org/10.48550/arXiv.1412.6980 (2017).

13. Song, J.-H., van de Groep, J., Kim, S. J. & Brongersma, M. L. Non-local metasurfaces for spectrally decoupled wavefront manipulation and eye tracking. *Nat. Nanotechnol.* **16**, 1224–1230 (2021).

14. Li, Y., Semmen, J., Yang, Q. & Wu, S.-T. Switchable polarization volume gratings for augmented reality waveguide displays. *J Soc Inf Display* **31**, 328–335 (2023).
